# Trends in Postpartum Depression by Race, Ethnicity, and Prepregnancy Body Mass Index

**DOI:** 10.1001/jamanetworkopen.2024.46486

**Published:** 2024-11-20

**Authors:** Nehaa Khadka, Michael J. Fassett, Yinka Oyelese, Nana A. Mensah, Vicki Y. Chiu, Meiyu Yeh, Morgan R. Peltier, Darios Getahun

**Affiliations:** 1 Department of Research and Evaluation, Kaiser Permanente Southern California, Pasadena; 2 Obstetrics and Gynecology, Kaiser Permanente, Los Angeles, California; 3 Department of Obstetrics and Gynecology, Beth Israel Deaconess Medical Center, Boston, Massachusetts; 4 Now with Brigham Young University, Provo, Utah; 5 Department of Psychiatry and Behavioral Health, Jersey Shore University Medical Center, Neptune, New Jersey; 6 Department of Psychiatry and Behavioral Health, Hackensack-Meridian School of Medicine, Nutley, New Jersey

## Abstract

**Importance:**

Postpartum depression (PPD) poses significant risks to maternal and child health. Understanding temporal trends is crucial for evaluating prevalence and identifying populations at risk.

**Objective:**

To evaluate recent trends in PPD and assess how these trends are associated with race, ethnicity, and prepregnancy body mass index (BMI).

**Design, Setting, and Participants:**

A serial, cross-sectional analysis using data from the Kaiser Permanente Southern California (KPSC) electronic health records (EHRs), with live and stillbirths at 20 or more weeks of gestation who were KPSC members at the time of delivery between 2010 and 2021. Data were analyzed from July 2022 to August 2023.

**Exposures:**

Self-reported race, ethnicity, and recorded prepregnancy BMI.

**Main outcome measures:**

PPD cases were identified using validated diagnostic codes and prescription records within 12 months postpartum in the KPSC EHRs. Patients with an Edinburgh Postnatal Depression Scale score of 10 or more within 6 months postpartum were further evaluated by a mental health specialist for formal PPD diagnosis.

**Results:**

In this study of 442 308 pregnancies, the median (IQR) maternal age at delivery was 31 (27-34) years. The cohort was racially and ethnically diverse, with 62 860 individuals (14.2%) identifying as Asian/Pacific Islander, 231 837 (52.4%) as Hispanic, 33 207 (7.5%) as non-Hispanic Black, 108 201 (24.5%) as non-Hispanic White, 5903 (1.3%) as multiple or other, and 300 (0.1%) unknown. PPD prevalence doubled over the study period, increasing from 9.4% in 2010 to 19.0% in 2021. The largest increases were observed among Asian and Pacific Islander participants (280% increase) and non-Hispanic Black participants (140% increase). PPD rates increased across all BMI categories, particularly among individuals with obesity (class I) and morbid obesity (class II/III).

**Conclusions and Relevance:**

In this cross-sectional study, PPD diagnosis increased significantly across all racial and ethnic groups and BMI categories over the past decade. While rising PPD may reflect improved screening and diagnosis practices, the persistently high rates highlight the need to develop and implement interventions to prevent the condition while expanding efforts to mitigate the impact of PPD on maternal and child health.

## Key Points

**Question:**

What are the trends in postpartum depression (PPD) over the past decade, and how do PPD trends differ by maternal race, ethnicity, and prepregnancy body mass index?

**Findings:**

In this serial cross-sectional study of 442 308 births, a significant rise in PPD diagnosis rates was observed across all racial and ethnic groups, increasing from 9.4% in 2010 to 19.0% in 2021. The prevalence of PPD increased with higher prepregnancy body mass index, highlighting the importance of considering maternal health factors in PPD risk assessment.

**Meaning:**

These findings suggest that the prevalence of PPD is high and rising, underscoring the need for continued research to better prevent, diagnose, treat, and mitigate the impact of this condition.

## Teaser

This cross-sectional study evaluates how recent trends in postpartum depression are associated with race, ethnicity, and prepregnancy body mass index.

## Introduction

Postpartum depression (PPD) is a depressive disorder that occurs within 12 months following childbirth.^1^ The prevalence of PPD ranges between 10% and 20% globally^2^ and is about 13% in the US,^3^ making it one of the most common adverse pregnancy outcomes.^4^ Characterized by persistent feelings of sadness, anxiety, and loss of interest or pleasure in activities, untreated PPD can impact both the mother and child. Maternal outcomes include increased maternal morbidity,^5^ lower rates of breastfeeding,^6^ and impaired maternal-infant bonding,^7^ while children face higher rates of developmental delays.^7^ In severe cases, untreated PPD can lead to suicide or infanticide,^8^ underscoring the urgency of addressing this condition. Furthermore, PPD has been associated with long-term neurodevelopmental effects in children that are not apparent at birth, such as an increased risk of autism spectrum disorders^9^ and attention-deficit/hyperactivity disorder.^10^

Racial and ethnic disparities are evident in the disease burden of PPD, with Black mothers experiencing the highest prevalence of the disorder.^11^ Race and ethnicity are key factors influencing maternal health during pregnancy, including the risk of PPD.^11^ Understanding how PPD rates vary by race and ethnicity is important for identifying high-risk groups and addressing inequities in maternal mental health care. Research has demonstrated that other factors, such as high prepregnancy body mass index (BMI),^12^ neighborhood disadvantage,^11^ and long-term exposure to air pollution^11^ also contribute to the increased risk of PPD.

Recognizing the importance of early detection and intervention, health care systems have increasingly integrated PPD screenings into routine postpartum and well-child visits. The American College of Obstetricians and Gynecologists recommends comprehensive screening for PPD during postpartum visits using validated instruments, such as the Edinburgh Postnatal Depression Scale (EPDS).^13^ Similarly, the American Academy of Pediatrics recommends integrating PPD screening at 1 to 2-, 4-, and 6-month well-child visits.^14^ However, despite these initiatives, PPD continues to be an underdiagnosed and undertreated adverse perinatal outcome.

Previous analyses by the US Centers for Disease Control and Prevention (CDC) indicated a declining prevalence of postpartum depressive symptoms from 2004 (15%) to 2012 (10%), but this study excluded California data and relied on self-reports rather than clinically diagnosed cases. Thus, this study aims to elucidate temporal trends in PPD prevalence by race, ethnicity, and prepregnancy BMI.

## Methods

This study followed the Strengthening the Reporting of Observational Studies in Epidemiology (STROBE) reporting guideline for cross-sectional studies^15^ and was performed with the approval and oversight of the Kaiser Permanente Southern California (KPSC) institutional review board with a waiver of informed consent because data were deidentified.

Data from electronic health records (EHRs) at KPSC were used to conduct this serial cross-sectional study. KPSC is a large integrated health care delivery system servicing care to over 4.8 million members across 15 medical centers and 236 medical offices.^16^ The demographic composition of KPSC members reflects the diversity of the Southern California region.^17^ KPSC’s EHR system encompasses comprehensive information on member patients receiving in- and outpatient care, including diagnostic and procedural codes, pharmacy and laboratory records, and member demographics and behavioral information.

For this analysis, we identified all live and stillbirths at 20 or more weeks of gestation between January 1, 2010, and December 31, 2021. Eligibility for inclusion in the study required KPSC membership at the time of pregnancy delivery.

### Outcome

PPD was defined as the presence of a depressive disorder diagnosis according to the *International Classification of Diseases, Ninth and Tenth Revision (ICD-9 *and* ICD-10)* diagnostic codes provided by a mental health specialist and/or by use of antidepressants prescribed for PPD within 12 months following childbirth (eTable 1 in Supplement 1).

PPD diagnosis at KPSC follows a 2-step process. First, depression status is routinely assessed during well-child visits for postpartum individuals; over 95% of individuals with KPSC pregnancies with live birth deliveries were assessed for their depression status during the first 6 months of well-child visits. Then, those exhibiting signs and symptoms of depression undergo further evaluation using standardized questionnaires, such as the EPDS, administered by social workers. Individuals who score 10 or higher on the EPDS are referred for a clinical interview with mental health professionals,^13^ who conduct a comprehensive assessment and provide follow-up care, diagnosis, and treatment.^18^ Our recent validation study demonstrated that supplementing diagnostic codes with pharmacy use records significantly enhanced the completeness and accuracy of PPD case identification (sensitivity, 98.3%; specificity, 83.3%; positive predictive value, 93.7%; negative predictive value, 95.0%) compared with relying solely on EPDS/Patient Health Questionnaire-9 surveys or diagnostic codes.^19^

### Covariates

BMI was calculated as weight in kilograms divided by height in meters squared and categorized into 5 groups: individuals who were underweight (<18.5), normal weight (18.5–24.9), overweight (25.0–29.9), obese class I (30–34.9), and obese class II/III (≥35.0). Self-reported race and ethnicity data were extracted from EHRs and categorized as non-Hispanic White, non-Hispanic Black, Hispanic, Asian and Pacific Islander, and Other and multiple groups (multiple or other refers to those with more than 1 race and/or ethnicity and/or non-Hispanic American Indian or Alaska Native groups). Race and ethnicity were assessed in this study to provide race and ethnicity-specific rates for PPD and to identify potential disparities in mental health disorders.

Additional covariates included maternal age at delivery (<20, 20-29, 30-34, and ≥35 years), education level (less than high school, high school graduate, some college, associate/bachelor’s degree, and master’s or above), parity (nulliparous or multiparous), timing of prenatal care initiation (≤3 months or late/no care), smoking status during pregnancy, alcohol use during pregnancy, and median family household income. Annual median household income was estimated based on census tract information with inflation adjustment and classified into 5 categories: $29 999 or less; $30 000 to $49 999; $50 000 to $69 999; $70 000 to $89 999; and $90 000 or more. Missing data were labeled as unknown for race and ethnicity and missing for all other covariates.

### Statistical Analysis

The distribution of selected characteristics was stratified by PPD status and evaluated using appropriate statistical tests, including χ^2^ (categorical variables) and independent *t* tests (continuous variables). Pregnancy-related covariates and potential confounders were selected based on a priori considerations and were either accounted for using stratified analysis or by adjustment in the models. Missing observations were reported in the tables and accounted for in the models.

To assess trends over time, we calculated the annual rates of PPD, then modeled the stratified datasets using modified Poisson regression approach with a robust error variance for binary outcomes to quantify the linear trends for PPD while accounting for potential confounding factors and nonindependence of records that may occur due to multiple deliveries from the same patient during the study period.^20^ The outcome in this study was dichotomous (PPD vs no PPD) and assessed within the first 12 months post partum for all participants. Additionally, we examined crude changes in the proportion of PPD between the earliest (2010) and most recent (2021) periods, stratified by race, ethnicity, and prepregnancy BMI. Moreover, we performed stratified analysis by examining the temporal trends of PPD with diagnosis at 42, 90, and 180 days compared with 2010 (the reference year). As a sensitivity analysis, we examined trends of PPD after excluding those with depression during pregnancy (antepartum depression). Furthermore, we also assessed whether there was a 3-way interaction between year of delivery, maternal race and ethnicity, and prepregnancy BMI. Results are also presented as adjusted risk ratios (RR) with 95% CIs for linear trends. Results where a 2-sided *P *value was less than .05 were considered statistically significant. All statistical analyses were performed using SAS statistical software, version 9.4 (SAS Institute, Inc). Data were analyzed from July 2022 to August 2023.

## Results

Among the 442 308 individuals in this study, the median (IQR) maternal age at delivery was 31.0 (27.0-34.0) years. The cohort was racially and ethnically diverse, with 62 860 individuals (14.2%) identifying as Asian/Pacific Islander, 231 837 (52.4%) as Hispanic, 33 207 (7.5%) as non-Hispanic Black, 108 201 (24.5%) as non-Hispanic White, 5903 (1.3%) as multiple or other, and 300 (0.1%) unknown. The majority had an associate’s degree, bachelor’s degree, or higher (192 915 individuals [57.3%]); were multiparous (248 350 individuals [56.1%]); and initiated prenatal care during the first trimester (370 348 individuals [83.7%]).

Table 1 presents the distribution of characteristics stratified by PPD status. In our cohort, 61 556 individuals (13.9%) had a PPD diagnosis and/or received a prescription for antidepressants within 12 months of childbirth between 2010 and 2021. Compared with individuals without a PPD diagnosis, PPD was more frequently diagnosed among those who were older, with 21 577 individuals aged 30 to 34 years (35.1%) compared with 126 887 (33.3%) in the non-PPD group, and 16 781 individuals aged 35 years or older (27.3%) compared with 88 099 (23.1%) in the non-PPD group. We further examined PPD trends with diagnosis at 42, 90, and 180 days compared with 2010 (the reference year) as a sensitivity analysis and found that the trend persisted (eTable 2 in Supplement 1). When the follow-up was limited to 42 days after delivery, the relative increase in PPD trend over time was 3.17-fold (95% CI, 2.92-3.45). Similarly, when the follow-up was limited to 90 days after delivery, the relative increase in PPD trend over time was 2.80-fold (95% CI, 2.64-2.97).

**Table 1.** Distribution of Maternal Characteristics by Postpartum Depression (PPD) Status^a^

Characteristic	Patients, No. (%)	
	No PPD (n = 380 752)	PPD (n = 61 556)
Age at delivery, y		
Median (IQR)	30.0 (26.0-34.0)	31.0 (27.0-35.0)
<20	11 674 (3.1)	1191 (1.9)
20-29	154 092 (40.5)	22 007 (35.8)
30-34	126 887 (33.3)	21 577 (35.1)
≥35	88 099 (23.1)	16 781 (27.3)
Race and ethnicity		
Asian/Pacific Islander	57 674 (15.1)	5186 (8.4)
Hispanic	199 724 (52.5)	32 113 (52.2)
Non-Hispanic Black	27 967 (7.3)	5240 (8.5)
Non-Hispanic White	90 140 (23.7)	18 061 (29.3)
Multiple or other^b^	5009 (1.3)	894 (1.5)
Unknown	238 (0.1)	62 (0.1)
Education		
Less than HS	16 921 (4.4)	1695 (2.8)
HS graduate	80 178 (21.1)	11 702 (19.0)
Some college	74 062 (19.5)	13 176 (21.4)
Associate/bachelor’s degree	115 566 (30.4)	16 988 (27.6)
Master’s degree/above	52 054 (13.7)	8307 (13.5)
Missing	41 971 (11.0)	9688 (15.7)
Parity		
Multiparous	212 369 (55.8)	35 981 (58.5)
Nulliparous	114 216 (30.0)	18 520 (30.1)
Missing	54 167 (14.2)	7055 (11.5)
Initiation of prenatal care		
≥3 mo	316 996 (83.3)	53 352 (86.7)
Late/no care	57 706 (15.2)	7072 (11.5)
Missing	6050 (1.6)	1132 (1.8)
Smoked during pregnancy	8556 (2.2)	2349 (3.8)
Alcohol use during pregnancy	47 916 (12.6)	11 042 (17.9)
Median household income, US $^c^		
<30 000	8170 (2.1)	948 (1.5)
30 000-49 999	81 062 (21.3)	11 506 (18.7)
50 000-69 999	109 201 (28.7)	17 624 (28.6)
70 000-89 999	84 781 (22.3)	14 617 (23.7)
≥90 000	96 809 (25.4)	16 780 (27.3)
Missing	729 (0.2)	81 (0.1)
Prepregnancy BMI^d^		
<18.5	7690 (2.0)	819 (1.3)
18.5-24.9	139 172 (36.6)	19 159 (31.1)
25.0-29.9	94 008 (24.7)	16 200 (26.3)
30.0-34.9	51 492 (13.5)	10 287 (16.7)
≥35.0	39 508 (10.4)	9455 (15.4)
Missing	48 882 (12.8)	5636 (9.2)

Abbreviations: HS, high school; BMI, body mass index.

^a^ Differences between PPD status by maternal characteristics were statistically significant (all *P* < .001).

^b^ Multiple or other refers to those with more than 1 race and/or ethnicity and/or non-Hispanic American Indian or Alaska Native groups.

^c^ Annual median household income based on census tract information with inflation adjustment.

^d^ Calculated as weight in kilograms divided by height in meters squared.

Compared with individuals without a PPD diagnosis, those with a PPD diagnosis were more likely to be non-Hispanic White (18 061 [29.3%] vs 90 140 individuals [23.7%]) and non-Hispanic Black (5240 [8.5%] vs 27 967 individuals [7.3%]). Additionally, individuals with PPD were more likely to be multiparous (35 981 [58.5%] vs 212 369 individuals [55.8%]), to have smoked during pregnancy (2349 [3.8%] vs 8556 individuals [2.2%]), or used alcohol during pregnancy (11 042 [17.9%] vs 47 916 individuals [12.6%]). PPD was also more frequently diagnosed among those with overweight (16 200 [26.3%] vs 94 008 individuals [24.7%]) or obesity, with a higher prevalence in individuals with class I obesity (10 287 [16.7%] vs 51 492 individuals [13.5%]) and class II/III obesity (9455 [15.4%] vs 39 508 individuals [10.4%]).

**Figure 1.**
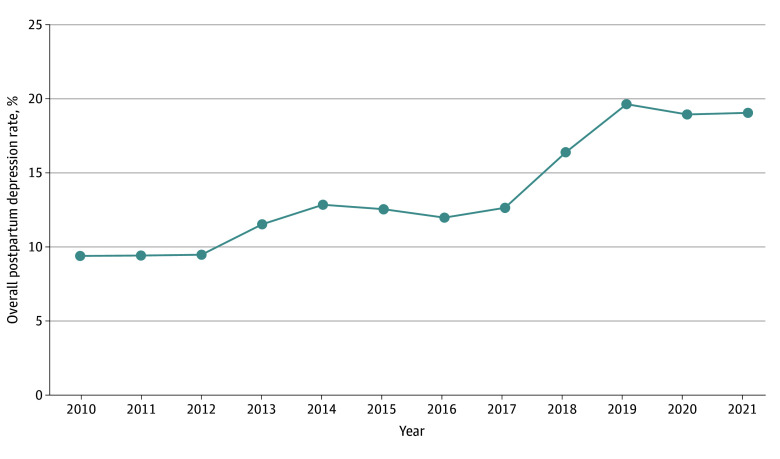
Overall Rates of Postpartum Depression in Kaiser Permanente Southern California

The overall PPD rates increased during the study period from 9.4% in 2010 to 19.0% in 2021 (*P* for trend < .001) (Figure 1). The largest relative increases of PPD were observed in the years 2013 (22% increase from 2012), 2018 (30% increase from 2017), and 2019 (20% increase from 2018). Table 2 presents the RRs and corresponding 95% CIs for the relative changes in PPD by race and ethnicity when comparing 2021 vs 2010. PPD rates increased for all races and ethnicities, with the highest relative increase observed among Asian and Pacific Islander individuals, rising from 3.6% in 2010 to 13.8% in 2021 (RR, 3.8; 95% CI, 3.2-4.5; *P* for trend < .001) (eFigure in Supplement 1). This was followed by rates among non-Hispanic Black individuals, which increased from 9.2% in 2010 to 22.0% in 2021 (RR, 2.4; 95% CI, 2.1-2.8; *P* for trend < .001), and Hispanic individuals, with rates increasing from 8.9% to 18.8% (RR, 2.1; 95% CI, 2.0-2.3; *P* for trend < .001). Additionally, PPD rates increased among other and multiple race and ethnic groups, from 11.3% to 19.1% (RR, 1.7; 95% CI, 1.2-2.4; *P* for trend < .001), and among non-Hispanic White individuals, from 13.5% to 21.8% (RR, 1.6; 95% CI, 1.5-1.7; *P* for trend < .001). The largest increases were observed among Asian and Pacific Islander participants (280% increase) and non-Hispanic Black participants (140% increase).

**Table 2.** Rates and Relative Increases in Postpartum Depression (PPD) Rates Among Kaiser Permanente Southern California Members by Maternal Race and Ethnicity, 2010-2021^a^

Race and ethnicity	Patients, No. (%)												Mean (SD) rate	Crude RR (95% CI) for 2021 vs 2010	Crude RR (95% CI) for race and ethnicity on overall PPD	Adjusted linear trends, *P* values^b^
	2010	2011	2012	2013	2014	2015	2016	2017	2018	2019	2020	2021				
Total births (n = 442 308)	30 460	32 335	34 321	34 382	35 486	37 188	38 685	39 016	40 033	40 297	39 856	40 249	NA	NA	NA	NA
PPD (n = 61 556)	2862 (9.4)	3047 (9.4)	3253 (9.5)	3964 (11.5)	4553 (12.8)	4660 (12.5)	4630 (12.0)	4930 (12.6)	6558 (16.4)	7904 (19.6)	7539 (18.9)	7656 (19.0)	13.9 (3.9)	2.02 (1.95-2.11)	NA	<.001
Rates of PPD																
Asian/Pacific Islander (n = 5186)	145 (3.6)	179 (4.1)	194 (3.9)	290 (6.2)	372 (7.5)	371 (7.1)	297 (5.5)	370 (6.6)	546 (9.3)	816 (13.5)	812 (13.9)	794 (13.8)	8.3 (3.9)	3.81 (3.21-4.53)	0.47 (0.45-0.49)	<.001
Hispanic (n = 32 113)	1405 (8.9)	1529 (9.2)	1595 (9.2)	2027 (11.6)	2327 (12.6)	2394 (12.3)	2430 (11.9)	2581 (12.5)	3543 (16.6)	4281 (20.0)	4003 (18.4)	3998 (18.8)	13.9 (4.0)	2.12 (2.00-2.25)	0.85 (0.83-0.86)	<.001
Non-Hispanic Black (n = 5240)	244 (9.2)	259 (9.6)	280 (10.4)	336 (12.2)	390 (14.3)	402 (14.5)	387 (13.4)	426 (15.3)	608 (21.6)	655 (23.4)	632 (22.6)	621 (22.0)	15.8 (5.3)	2.40 (2.09-2.76)	0.98 (0.95-1.01)	<.001
Non-Hispanic White (n = 18 061)	1037 (13.5)	1053 (12.7)	1156 (12.8)	1273 (13.9)	1414 (15.7)	1439 (15.7)	1438 (15.1)	1489 (15.8)	1738 (18.5)	2015 (21.5)	1966 (22.3)	2043 (21.8)	16.7 (3.5)	1.62 (1.51-1.73)	1 [Reference]	<.001
Other/multiple (n = 894)	31 (11.3)	27 (9.0)	28 (8.0)	38 (10.3)	49 (12.9)	52 (11.5)	78 (14.8)	64 (12.6)	123 (18.9)	136 (20.8)	124 (18.3)	144 (19.1)	15.1 (4.3)	1.69 (1.18-2.43)	0.90 (0.84-0.97)	<.001

Abbreviations: NA, not applicable; RR, risk ratio.

^a^ Unknown race was excluded due to small samples.

^b^ Adjustments were made for maternal age, median household income, parity, prenatal care, smoking during pregnancy, alcohol use during pregnancy, and prepregnancy BMI.

**Figure 2.**
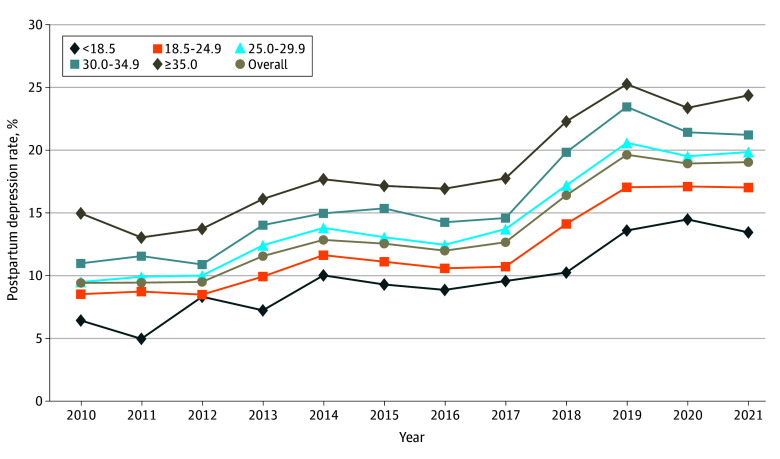
Prepregnancy Body Mass Index–Specific Rates of Postpartum Depression in Kaiser Permanente Southern California Adjustments were made for maternal age, race and ethnicity, median household income, parity, prenatal care, smoking during pregnancy, and alcohol use during pregnancy.

Figure 2 displays the rates of PPD by prepregnancy BMI across the study period. PPD rates increased for all prepregnancy BMI groups between 2010 and 2021 (from 6.4% to 13.4% among those with underweight; *P* for trend <.001; 8.5% to 17.0% among those with normal weight; *P* for trend < .001; from 9.5% to 19.8% among those with overweight; *P* for trend < .001; 11.0% to 21.2% among those with class I obesity; *P* for trend < .001; and 14.9% to 24.4% among those with class II/III obesity; *P* for trend < .001). Compared with the baseline year of 2010, the RR for PPD at 2021 was 2.1 (95% CI, 1.5-3.0; *P* for trend < .001) for the group with underweight, 2.0 (95% CI, 1.9-2.2; *P* for trend < .001) for the group with normal weight 2.1 (95% CI, 1.9-2.3; *P* for trend < .001) for the group with overweight, 1.9 (95% CI, 1.8-2.2; *P* for trend < .001) for the group with obesity class I, and 1.6 (95% CI, 1.5-1.8; *P* for trend < .001) for the group with obesity class II/III. The prevalence of PPD has been rising over the past decade, alongside a parallel increase in prepregnancy BMI, with individuals having the highest prepregnancy BMI experiencing the highest rates of PPD throughout the study period. The 3-way interaction of PPD by year of delivery, maternal race and ethnicity, and prepregnancy BMI had a *P* value of .94, suggesting that the trend is likely to be similar for different BMI groups across the race and ethnicity groups. Sensitivity analysis excluding antepartum depression from the PPD cases revealed no changes from our overall findings.

## Discussion

Our study estimated PPD prevalence trends within a large obstetric cohort of 442 308 individuals over a decade-long period (2010-2021) using EHR data from the KPSC health care system. The findings demonstrated a 2-fold increase in the prevalence of PPD during the study period, with the largest increases in 2013, 2018, and 2019. Several factors may explain this trend: (1) the actual rise in PPD incidence, (2) increased awareness and improved surveillance of PPD, or (3) changes in screening and diagnostic practices, particularly following the transition from *ICD-9* to *ICD-10* in 2015 to 2016 within KPSC EHRs. Meanwhile, the rise in PPD rates between 2018 and 2019 coincided with the implementation of California Maternal Mental Health Screening Laws (Assembly Bills 2193 and 3032),^21,22^ which required obstetric clinicians to provide universal screening for maternal mental health conditions, including PPD, by the beginning of July 2019 and required health plans to develop educational programs for postpartum mental health. Moreover, there were new HEDIS measures^23^ (a tool used by health plans nationally to measure their care and service performance) introduced in 2020 that focused on improving postpartum depression screenings and follow-up.

While our findings of rising PPD are consistent with other studies reporting increased mental health disorders among US adults,^24,25^ they contrast with findings from a prior trend analysis by the CDC^26^ that suggested a decline in postpartum depressive symptoms. These discrepancies may be explained by differences in data collection methods. The CDC analysis used a self-reported measure for depression using a 2-item screening tool based on the Patient Health Questionnaire-2; these tests often have low sensitivity (58%) compared with clinical assessments.^27^ Meanwhile, our study relied on clinically confirmed diagnoses using validated tools such as the EPDS. The CDC publication was also limited because it only reported postpartum depressive symptoms for a subset of US states that did not include California, the most populous and ethnically diverse state in the nation. In our analysis, we found heterogeneity in the PPD trend by race and ethnicity, which is an important factor to consider when generalizing findings to the broader US population. Specifically, we observed that Asian and Pacific Islander individuals exhibited the largest increase in PPD diagnosis rates, with a 3.8-fold increase (95% CI, 3.2-4.5). This finding underscores the potential variability in PPD trends across different racial and ethnic groups, a factor that may not be fully captured in nationwide analyses, such as the CDC analysis, that excluded California.

In addition to temporal trends, we identified several demographic and clinical factors that likely increased the PPD prevalence. Women of advanced maternal age, those identifying as non-Hispanic White or non-Hispanic Black race and ethnicity, individuals reporting substance use during pregnancy, and those with higher prepregnancy BMI levels had a higher prevalence of PPD. These findings align with prior research highlighting the importance of tailored interventions to address the mental health needs of these high-risk groups.^11,28,29,30,31^

Furthermore, our study highlights that while disparities in PPD rates across racial and ethnic groups were narrowing, rising trends were observed across all races and ethnicities with non-Hispanic White and non-Hispanic Black groups exhibiting the highest rates of PPD diagnosis in 2021. Disparities in PPD care have been well documented, particularly among Black and Latina women, who may face barriers to treatment initiation.^32^ Asian and Pacific Islander individuals were more likely to receive a diagnosis for depression after clinicians initiated the conversation, but were far less likely to start this conversation with their clinicians compared with other groups.^29^ Social support has been identified as a critical protective factor across all racial and ethnic groups, underscoring the importance of culturally sensitive interventions that foster social support networks during the postpartum period.^33^

Furthermore, our analysis highlights the association between BMI and PPD risk, with consistently higher rates of PPD observed among individuals with higher prepregnancy BMI levels. These findings contribute to the existing literature on the association between maternal obesity and perinatal depression,^30,34,35,36^ highlighting the need for targeted interventions aimed at supporting the mental health of individuals with elevated BMI during pregnancy and postpartum.

### Strengths and Limitations

Our study had several strengths. First, we used a large, sociodemographically diverse cohort of over 442 000 individuals, which increases the robustness and generalizability of our findings, especially for populations of varying racial, ethnic, and socioeconomic backgrounds. Additionally, the use of KPSC’s comprehensive EHRs allowed for the identification of PPD cases using validated methods^19^ through a combination of clinical diagnostic codes and pharmacy records, enhancing the accuracy of PPD case identification. Our data, spanning over a decade, allowed us to capture meaningful trends in PPD prevalence over time, contributing valuable insights to the literature on maternal mental health. Moreover, the standardized screening practices employed by KPSC, including the use of validated instruments such as the EPDS, ensured consistent identification and diagnosis of PPD across the health care system. This uniformity in screening practices strengthens the reliability of the observed trends and associations.

Our findings, however, are not without limitations. First, we may have underestimated the true prevalence of PPD, as not all cases may have been captured through clinical screening or postpartum visits. Prior research suggests that depression is underdiagnosed and undertreated with about 60% of those with depressive symptoms never receiving a clinical diagnosis and even fewer receiving treatment.^26^ While our analysis used detailed health records from the KPSC system, care received outside the system may not have been captured if it was not submitted for reimbursement at KPSC, which would result in missed diagnoses and underestimation of the true prevalence rate of the condition. Moreover, the generalizability of our results is limited to populations receiving care within integrated health care systems like KPSC. The study did not account for potential variations in access to care or health care utilization patterns outside such systems.

## Conclusions

In this cross-sectional study, we observed a significant increase in PPD diagnosis over the 2010 to 2021 period across all racial and ethnic groups, as well as all BMI categories. Rising PPD diagnoses may be a result of improved screening and diagnosis practices. However, the high burden of PPD underscores the need for enhanced treatment interventions to improve maternal mental health outcomes. Targeted efforts to address racial and ethnic disparities and the mental health needs of high-risk groups, including women with elevated prepregnancy BMI, may help mitigate the impact of PPD on maternal and child well-being. Integrating behavioral health services within primary care, extending Medicaid coverage through the postpartum period, and providing reimbursement for PPD screening at well-baby visits may enhance postnatal mental health care delivery. Our study emphasizes the need for continued research and closely monitoring the rising trends of PPD. By identifying trends of PPD and associated demographic and clinical factors, our findings provide valuable insights for guiding future public health initiatives aimed at improving perinatal mental health outcomes and promoting maternal and child well-being.

## Notes

Supplement 1. **eTable 1. **List of *International Statistical Classification of Diseases and Related Health Problems, Ninth and Tenth Revision *(*ICD-9 *and *ICD-10*) Depression Diagnosis Codes and Medication List Used for Postpartum Depression Case Ascertainment **eFigure. **Maternal Race and Ethnicity-Specific Rates of Postpartum Depression (PPD) in Kaiser Permanente Southern California (2010-2021) **eTable 2. **Postpartum Depression (PPD) by the Time of Diagnosis

Supplement 2.
Data Sharing Statement
