# Supplementary material for: Trends in Postpartum Depression by Race, Ethnicity, and Prepregnancy Body Mass Index
Source: JAMA Netw Open. 2024 Nov 20;7(11):e2446486. doi: 10.1001/jamanetworkopen.2024.46486 (PMC11579791; doi:10.1001/jamanetworkopen.2024.46486)
Supplement: Supplement 1. — eTable 1. List of International Statistical Classification of Diseases and Related Health Problems, Ninth and Tenth Revision (ICD-9 and ICD-10) Depression Diagnosis Codes and Medication List Used for Postpartum Depression Case Ascertainment eFigure. Maternal Race and Ethnicity-Specific Rates of Postpartum Depression (PPD) in Kaiser Permanente Southern California (2010-2021) eTable 2. Postpartum Depression (PPD) by the Time of Diagnosis [file jamanetwopen-e2446486-s001.pdf]

Supplemental Online Content

Khadka N, Fassett MJ, Oyelese Y, et al. Trends in postpartum depression by race, ethnicity, and prepregnancy body mass index. *JAMA Netw Open*. 2024;7(11):e2446486. doi:10.1001/jamanetworkopen.2024.46486

eTable 1. List of *International Statistical Classification of Diseases and Related Health Problems, Ninth and Tenth Revision* (*ICD-9* and *ICD-10*) Depression Diagnosis Codes and Medication List Used for Postpartum Depression Case Ascertainment
eFigure. Maternal Race and Ethnicity-Specific Rates of Postpartum Depression (PPD) in Kaiser Permanente Southern California (2010-2021)
eTable 2. Postpartum Depression (PPD) by the Time of Diagnosis

This supplemental material has been provided by the authors to give readers additional information about their work.

© 2024 Khadka N et al. *JAMA Netw Open.*

eTable 1. List of *International Statistical Classification of Diseases and Related Health Problems, Ninth and Tenth Revisions* (*ICD-9* and *ICD-10*) Depression Diagnosis Codes and Medication List Used for Postpartum Depression Case Ascertainment

ICD-9 Codes	ICD-10 Codes	Medication List
300.4, 309.0, and 311	F32.9, F33.0, F33.2, F33.3, F33.41, F33.9, F34.1, F43.21, and F53.0	bupropion, Celexa, citalopram, Cymbalta, desvenlafaxine, duloxetine, Effexor, escitalopram, fluoxetine, Lexapro, paroxetine, Paxil, Pristiq, Prozac, sertraline, venlafaxine, Wellbutrin, and Zoloft.

eFigure. Maternal Race and Ethnicity-Specific Rates of Postpartum Depression (PPD) in Kaiser Permanente Southern California (2010-2021)

© 2024 Khadka N et al. *JAMA Netw Open.*

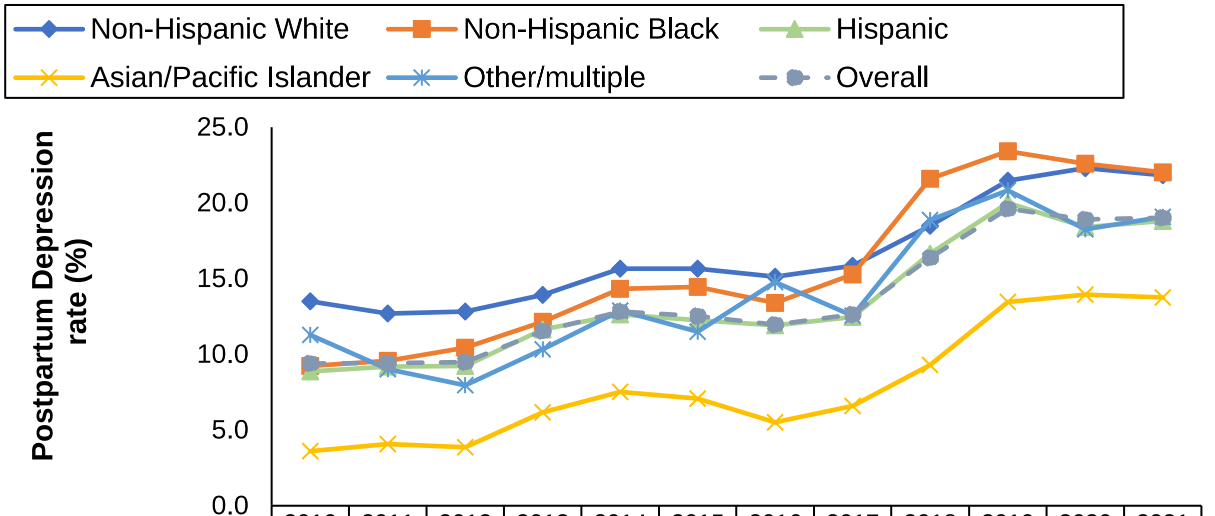

	2010	2011	2012	2013	2014	2015	2016	2017	2018	2019	2020	2021
Non-Hispanic White	13.5	12.7	12.8	13.9	15.7	15.7	15.1	15.8	18.5	21.5	22.3	21.8
Non-Hispanic Black	9.2	9.6	10.4	12.2	14.3	14.5	13.4	15.3	21.6	23.4	22.6	22.0
Hispanic	8.9	9.2	9.2	11.6	12.6	12.3	11.9	12.5	16.6	20.0	18.4	18.8
Asian/Pacific Islander	3.6	4.1	3.9	6.2	7.5	7.1	5.5	6.6	9.3	13.5	13.9	13.7
Other/multiple	11.3	9.0	8.0	10.3	12.9	11.5	14.8	12.5	18.9	20.8	18.3	19.1
Overall	9.4	9.4	9.5	11.5	12.8	12.5	12.0	12.6	16.4	19.6	18.9	19.0

Year

Race and Ethnicity-specific adjusted rates of Postpartum Depression (PPD): Kaiser Permanente Southern California (2010-2021). Adjustments were made for maternal age, median household income, parity, pre-pregnancy BMI, prenatal care, smoking during pregnancy, alcohol during pregnancy.

© 2024 Khadka N et al. *JAMA Netw Open.*

eTable 2. Postpartum Depression (PPD) by the Time of Diagnosis

Year	Total Births N= 442308	PPD Prevalence by The Time of Diagnosis			
		42 days N= 18909 (%)	90 days N= 34055 (%)	180 days N= 43073 (%)	365 days N= 61556 (%)
**2010**	30460	675 (2.2)	1319 (4.3)	1848 (6.1)	2862 (9.4)
**2011**	32335	705 (2.2)	1457 (4.5)	1976 (6.1)	3047 (9.4)
**2012**	34321	726 (2.1)	1457 (4.3)	2037 (5.9)	3253 (9.5)
**2013**	34382	1294 (3.8)	2168 (6.3)	2713 (7.9)	3964 (11.5)
**2014**	35486	1626 (4.6)	2668 (7.5)	3269 (9.2)	4553 (12.8)
**2015**	37188	1430 (3.9)	2485 (6.7)	3144 (8.5)	4660 (12.5)
**2016**	38685	890 (2.3)	2040 (5.3)	2795 (7.2)	4630 (12.0)
**2017**	39016	971 (2.5)	2214 (5.7)	3017 (7.7)	4930 (12.6)
**2018**	40033	1665 (4.2)	3465 (8.7)	4455 (11.1)	6558 (16.4)
**2019**	40297	3207 (8.0)	5234 (13.0)	6105 (15.2)	7904 (19.6)
**2020**	39856	2889 (7.3)	4665 (11.7)	5651 (14.2)	7539 (18.9)
**2021**	40249	2831 (7.0)	4883 (12.1)	6063 (15.1)	7656 (19.0)
**Average Rate**		4.3	7.7	9.7	13.9
**Crude RR (95% CI) for 2021 vs 2010**		3.17 (2.92, 3.45)	2.80 (2.64, 2.97)	2.48 (2.36, 2.61)	2.02 (1.95, 2.11)
**†Adjusted linear trends, p-values**		<.001	<.001	<.001	<.001

© 2024 Khadka N et al. *JAMA Netw Open.*
