# Supplementary material for: Trends in Postpartum Depression by Race, Ethnicity, and Prepregnancy Body Mass Index
Source: JAMA Netw Open. 2024 Nov 20;7(11):e2446486. doi: 10.1001/jamanetworkopen.2024.46486 (PMC11579791; doi:10.1001/jamanetworkopen.2024.46486)
Supplement: Supplement 2. — Data Sharing Statement [file jamanetwopen-e2446486-s002.pdf]

Data Sharing Statement

Khadka. Trends in Postpartum Depression by Race, Ethnicity, and Prepregnancy Body Mass Index. *JAMA Netw Open*. Published November 20, 2024.
doi:10.1001/jamanetworkopen.2024.46486

Data
Data available: No
